# Epinephrine stimulates CXCL1 IL‐1*α*, IL‐6 secretion in isolated mouse limb muscle

**DOI:** 10.14814/phy2.13519

**Published:** 2017-11-30

**Authors:** Alex J. Mattingly, Orlando Laitano, Thomas L. Clanton

**Affiliations:** ^1^ Department of Applied Physiology & Kinesiology University of Florida College of Health and Human Performance Gainesville Florida

**Keywords:** Deferoxamine, epinephrine, IL‐1alpha, keratinocyte chemoattractant, myokine, stress

## Abstract

Catecholamines stimulate interleukin‐6 (IL‐6) secretion in skeletal muscles. However, whether other cytokines are secreted is currently unknown. Skeletal muscle ex vivo preparations commonly used to study cytokine secretion have dealt with limitations including auto‐oxidation of catecholamines. The use of metal chelators could be an alternative to avoid auto‐oxidation and allow catecholamines to be used at physiological doses. We exposed isolated soleus muscles to 1 or 100 ng/mL epinephrine (EPI) and collected bath samples at 1 and 2 h for multiplex cytokine analysis. Keratinocyte chemoattractant (CXCL1), IL‐6, and IL‐1*α* were significantly elevated by 100 ng/mL exposure, but not by 1 ng/mL (median [CXCL1] (2 h) = 83 pg/mL; [IL‐6] = 19 pg/mL; IL‐1*α* = 7.5 pg/mL). CXCL1 and IL‐6 were highly correlated in each sample (*P* = 0.0001). A second experiment combined the metal chelator, deferoxamine mesylate (DFO), to prevent EPI autoxidation, with 2 ng/mL EPI and 10.5 ng/mL norepinephrine (NOREPI) to mimic peak exercise. Unexpectedly, DFO alone stimulated both IL‐6 and CXCL1 secretion, but together with EPI and NOREPI had no additional effects. Stimulation of cytokine secretory responses from skeletal muscle cells in response to DFO thus precludes its use as a chelating agent in ex vivo models. In conclusion, 100 ng/mL EPI stimulates a robust secretory CXCL1 response, which together with IL‐6 and IL‐1*α*, may constitute an adrenal‐muscle endocrine response system.

## Introduction

Stimulation of skeletal muscle cytokine secretion in response to catecholamine exposure has been of interest because of its potential contribution to plasma cytokine levels seen in intense exercise (Drenth et al. 1995), heat stress (Leon et al. 2006; King et al. 2017), and septic shock (Izeboud et al. 2004; Borge et al. 2009). These conditions have strong immunological responses and circulating catecholamines are elevated. Interleukin‐6 (IL‐6) has received the most attention because it is a predominant cytokine secreted by muscle during exercise and in many stress conditions (Pedersen and Febbraio 2008). However, increased skeletal muscle production of Interleukin‐1*β* (IL‐1*β*) and tumor necrosis factor‐*α* (TNF‐*α*) proteins have also been observed in response to *in vivo* epinephrine (EPI) infusion (Frost et al. 2004), which raises the question of whether the cytokine response is catecholamine‐specific or reflecting a more generalized activation of the innate immune cytokines.

While attempting to study the skeletal muscle responses to catecholamines in vitro, our group (Welc et al. 2016) and others (Holmes et al. 2004) have faced limitations inherent to the nature of the preparation. For instance, EPI is known to auto‐oxidize rapidly in physiological buffers (Ryan et al. 1993) which may underestimate the dose–response relationship. Techniques previously used to suppress auto‐oxidation using ascorbate were ineffective in oxygenated buffers (Sutor and ten Bruggencate 1990). In addition, what has been used to represent physiological levels of catecholamines during intense exercise were likely low, based on recently updated observations taken from blood, immediately after exercise in humans (Kröpfl et al. 2014). Therefore, to overcome these limitations our aims were as follows: (1) Prevent EPI autoxidation in vitro via buffer pretreatment with the metal chelator deferoxamine mesylate (DFO) (2) Use levels of EPI and NOREPI that were consistent with our current understanding of peak values achieved during intense exercise (Kröpfl et al. 2014).

Our results suggest that the cytokine secretory response to EPI in skeletal muscle is uniquely different from the classic cytokines released during the innate immune response, based largely on the absence of classic proinflammatory cytokines like TNF*α* and IL‐1*β*. However, the results also demonstrate for the first time, that at pharmacological doses of EPI, IL‐6 secretion is accompanied by robust secretion of a chemokine, CXCL1, commonly referred to as keratinocyte chemoattractant (KC), as well as by a transient elevation in interleukin‐1 alpha (IL‐1*α*).

## Methods

All protocols were approved by the University of Florida Institutional Animal Use Committee (IACUC# 201609372). Male C57Bl/6 male mice (25–30 g) were maintained in groups until they were transported to the laboratory from the animal vivarium the night before tissues were collected. All animals were kept on a 12:12‐h light‐dark cycle at 20–22°C/30–60% relative humidity (RH) and on a standard chow diet (LM‐485 m Envigo; Teklad, Madison, WI). Mice were anesthetized under isoflurane and their hind limbs removed. Solei (SOL) and, in some cases, extensor digitorum longus (EDL) muscles were excised rapidly under oxygenated (95% O_2_, 5% CO_2_) Krebs Ringers solution containing (in mmol/L) 0.45 Na_2_SO_4_, 0.6 Na_2_HPO_4_, 1.0 MgCl_2_, 5.9 KCl, 2.0 CaCl_2_, 21.0 NaHCO_3_, 121.0 NaCl, and 11.5 glucose. Muscles were then placed in temperature controlled 2 mL tissue baths (Radnoti 158303) at 35°C. The bath volumes were further reduced to 1 mL by placing glass rods around the outside of the muscle. Muscles were set at 1 g of tension which approximates the preload required to achieve optimal length in these muscles (Welc et al. 2012). Electrical stimulation of the muscles was avoided at all times because of the effects of contraction on cytokine production. The muscles were first allowed to equilibrate in the baths for a 30 min acclimation period.

Two sets of experiments were completed. In the first series, after acclimation, muscles were exposed to fresh buffer containing either 1 ng/mL (EPILOW) or 100 ng/mL (EPIHIGH) of L‐Epinephrine. The 100 ng/mL dose is generally considered a pharmacological dose, whereas the 1 ng/mL is within a range seen physiologically. The tissues (*N* = 8 in each group) remained undisturbed and samples were taken from the baths after 1 h (T1) or 2 h (T2) of incubation. A portion of this data (4% of the total) was published previously (Welc et al. 2016), namely, the EPI‐stimulated IL‐6 secretion at times T1 and T2. It was included in this study for comparison and correlation with other cytokines in the same samples. Bath samples were collected, mixed with protease inhibitor cocktail (Bimake, Houston TX), flash frozen in liquid nitrogen, and stored at −80°C prior to Luminex multiplex analysis using MILLIPLEX MAP Mouse cytokine/chemokine premixed 32‐plex assay kits (Sigma Millipore, St. Louis, MO). Of the 32 analytes, 18 of interest associated with the immune system were selected for investigation: IL‐6, KC, MIP‐2, IL‐15, GCS‐F, LIX, MIP‐1*α*, MIP‐1*β*, GM‐CSF, IP‐10, IL‐1 *α*, IL‐1b, IL‐12p70, IL‐10, IL‐12p40, TNF*α*, MCP‐1, and M‐CSF. The test was performed according to the manufacturer's protocols, as described elsewhere (Welc et al. 2012).

In the second series, an attempt was made to study cytokine secretion at more physiological levels of catecholamines (*N* = 16 per group). Muscles were exposed to a combination of L‐Epinephrine (2 ng/mL) and L‐Norepinephrine (10.5 ng/mL), corresponding to peak plasma levels seen in human exercise (Kröpfl et al. 2014). The buffers also contained 4 mmol/L DFO to reduce the rate of autoxidation of the EPI (Ryan et al. 1993). Muscles from eight mice were exposed to the DFO/EPI/NOREPI combination, with the other muscles being exposed to DFO control bath containing no catecholamines. L‐Epinephrine, L‐Norepinephrine, and deferoxamine mesylate were all obtained from Sigma Chemical, St. Louis, MO. The muscles were prevented from light exposure during incubation. Prior to exposure to DFO/catecholamine‐loaded buffer, these tissues were exposed to 30 min of fresh buffer and samples taken without stimulation. This data was used as the T0 time point and to obtain the baseline median and 25–75% quartiles for graphical representation. Matched experiments were performed in both SOL and EDL for each mouse in this group.

Statistical analyses were performed with SAS JMP Pro 12.2.0 (SAS Institute). The effect on accumulated cytokine protein excretion at T0, T1, and T2 were compared both between and within treatment groups via nonparametric Wilcoxin Kruskal‐Wallace test within groups, followed by the nonparametric Steel–Dwass post hoc test between groups. All significance tests were accompanied with a Benjamini–Hochberg test to account for false discovery rates with multiple testing. The Benjamini–Hochberg test was set at a false discovery rate threshold of <0.2. To determine differences in rates of secretion within specific time intervals, a paired *T* test was utilized because sample populations of matched differences were normally distributed. Correlations between responses of different cytokines were evaluated with linear regression and Pearson's correlation coefficient. Graphical relationships were created using GraphPad Prism (GraphPad Software).

## Results

**Figure 1:**
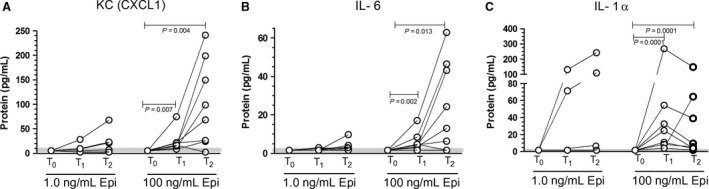
Changes in (A) keratinocyte chemoattractant, CXCL1, (B) Interleukin‐6 (IL‐6) and (C) Interleukin ‐1*α* in response to EPILOW (1 ng/mL) and EPIHIGH (100 ng/mL) treatments. *N* = 8 for each experiment, *P* values represent post hoc values. See Table 1 for Medians ±25–75% quartiles. Shaded line is centered on the median at the 25–75% quartiles for the baseline control.

Exposure to EPIHIGH significantly elevated CXCL1, IL‐6, and IL‐1*α* secretion into the tissue baths (Fig 1). The median CXCL1 exceeded the amount of IL‐6 secreted by four times (Table 1, Fig 1). For CXCL1 and IL‐6, the predominant secretion occurred during the second hour of exposure (T2‐T1) compared to the first (T1‐T0) (*P* < 0.05 for both cytokines). The IL‐1*α* response followed a different, more transient profile, with the peak levels in 7/8 of the samples being reached after only 1 h (median = 17.8) and then falling to a median of 7.84 by the 2‐h sample. There was also a very small statistically significant change in granulocyte colony‐stimulating factor (GCSF) in these samples (Table 1).

**Table 1:** Median responses ordered by significance in each experimental group

Treatment	Cytokine	Median _T2_	Quartiles _T2_	P _T0‐T2_	Q
EPIHIGH	IL‐1*α*	7.5	3.6–58.1	0.001	0.0126
	CXCL1	83.5	24.8–186.5	0.0015	0.0135
	GCSF^a^	2.6	2.5–8.0	0.003	0.0180
	IL‐6	18.6	2.8–45.6	0.0046	0.0207
EPILOW	MIP‐1*α*	8.1	7.8–10.0	0.0015	0.027
DFO	CXCL1	34.95	18.4–54.0	0.0001	0.0009
	IL‐6	3.49	2.6–9.6	0.0001	0.0009
	IP‐10^a^	2.15	1.9–2.7	0.0016	0.0096
DFO/EPI/NOREPI	IL‐6	4.36	2.2–20.8	0.0005	0.009

Data shown for differences between baseline and the 2 hr sample collection. Median and 25–75% Quartiles (Most sample populations were nonparametric). P = raw P from Kruskal Wallis Test/Wilcoxon post hoc test. Q = Benjamani–Hochberg adjusted *P* value for False Discovery Rate <0.2. Ordered by minimum Q value in each group.

EPI, epinephrine; NOREPI, norepinephrine; DFO, deferoxamine treatment

a Statistically significant but the median at T2 was <1 pg/mL greater than average T0 and therefore was considered to be biologically unimportant.

Exposure to EPILOW (1 ng/mL) for 2 h had no significant impact on CXCL1, IL‐6, or IL‐1*α* secretion from mouse soleus (Fig. 1A and B). MIP‐1*α* increased significantly to a median of ≈2.9 pg/mL above the average baseline. Since this small response did not appear at the higher dose of EPI, we attribute this to a nonspecific effect in these tissues.

**Figure 2:**
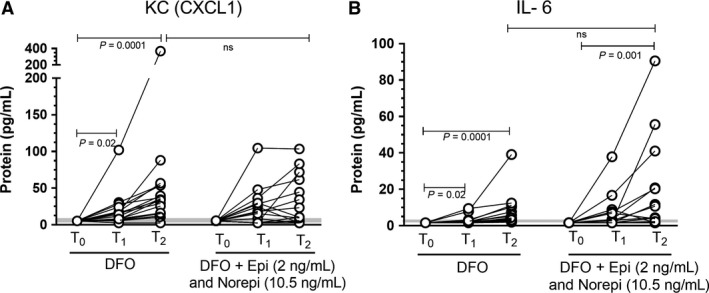
Changes in keratinocyte chemoattractant, CXCL1, and interleukin‐6 (IL‐6) to combined treatment with DFO or DFO+ EPI and norepinephrine (NOREPI). *N* = 15–16 in each group. *P* values within treatment groups are post hoc comparisons. Tests were also performed on differences in the amount of cytokine secreted at T2, in the control versus EPI/NOREPI treated tissues. See Table 1 for Medians ±25–75% quartiles. Shaded line is centered on the median at the 25–75% quartiles for the baseline control. EPI, epinephrine; DFO, deferoxamine.

In the second experiment using DFO with EPI and NOREPI, the DFO control baths caused a significant elevation in both CXCL1 and IL‐6 secretion (Fig. 2). When DFO was combined with EPI and NOREPI, there was no significant additional elevation in either CXCL1 or IL‐6, over and above what was observed by DFO treatment alone. Of the 18 cytokines of interest studied, only those noted above were observed to change significantly. No other cytokines were elevated in DFO + catecholamine treatment. No cytokines were significantly elevated in the matched baths containing EDL muscles.

**Figure 3:**
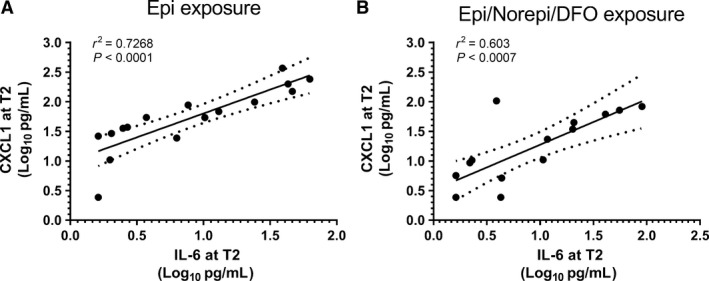
Linear regression and correlations between the amount of CXCL1 secreted and the amount of IL‐6 secreted for each tissue bath. Data transformed to a log‐log plot to improve the distribution. (A) experiments with EPILOW and EPIHIGH. (B) Experiments with combined epinephrine and norepinephrine in deferoxamine‐treated buffer. Dashed lines = 95% CI for the regressions.

Because of the clear resemblance of the proportional responses of CXCL1 and IL‐6, we tested whether CXCL1 and IL‐6 secretion were related by performing linear regression on individual measurements in each muscle exposed to catecholamines. As shown in Figure 3A and B, there were significant associations observed between IL‐6 and CXCL1 in each tissue studied and in both experiments in which muscles were exposed to EPI. No relationship between IL‐6 and CXCL1 with DFO treatment alone was observed, nor were there any significant relationships between the quantities of IL‐1*α* and IL‐6 secreted into the bath.

## Discussion

Our results demonstrate that a pharmacological dose (100 ng/mL) of EPI is a potent stimulus, not only of IL‐6, but also for CXCL1 and IL‐1*α* secretion in isolated mouse muscle. To our knowledge, this is the first report to demonstrate CXCL1 and IL‐1*α* secretion in isolated skeletal muscle in response to catecholamine stimulation. Though the elevation in IL‐1*α* is consistent with a generalized proinflammatory response, there was no evidence for increased TNF*α* or IL‐1*β* that were previously described for gastrocnemius muscle *in vivo* rat preparations (Frost et al. 2004). To put the amount of secretion in perspective, the average adult male mouse SOL muscle weighs ≈6 mg, or 0.6% of the volume of the bath. Adult mouse plasma volume is approximately 0.75 mL. Therefore, one SOL, under these conditions has the capacity to account for over 80 pg/mL CXCL1 in vivo.

We observed a close relationship between the quantities of IL‐6 and CXCL1 released in individual muscles exposed to EPI (Fig. 3). This suggests that these cytokines are either regulated in some way by similar transcriptional, translational, or secretory pathways or that secretion of one cytokine influences secretion of the other. Several lines of evidence suggest that IL‐6 can stimulate CXCL1 release in some phenotypes. For example, Pedersen et al. (2011) demonstrated that during exercise, liver CXCL1 mRNA expression is completely blocked in IL‐6 knockout mice and that overexpression of skeletal muscle IL‐6 resulted in elevations in CXCL1 expression during exercise. Similar types of findings have been reported in other cell and tissue systems (Ahuja et al. 2012; Roy et al. 2012). Despite the fact that the regulatory subunits for CXCL1 transcription contain a cyclic AMP response element, presumed to be responsive to *β*‐adrenergic stimulation [6], there is little evidence in the literature that catecholamines directly stimulate CXCL1 secretion on their own.

Perhaps a key to understanding both IL‐6 and CXCL1 responsiveness to catecholamines might be through understanding their interactions with IL‐1*α*. When brown fat (phenotypically similar to muscle, see editorial (Cesari 2008)) is exposed to any one of a variety of *β*‐adrenergic agonists at doses similar to those used in this study, they rapidly secrete IL‐6 and IL‐1*α* (Burýsek and Houstek 1997). IL‐1*α* is well known as a potent stimulator of IL‐6 through classic engagement with surface IL‐1 receptors, subsequent Nf‐*κ*B activation and phosphorylation of stress activated protein kinases (Weber et al. 2010). These same pathways can independently stimulate CXCL1 transcription (Amiri and Richmond 2003). In a model of inflammation during aging, levels of circulating and tissue IL‐6 and IL‐8 (a homologue of CXCL1) have been found to depend on cell surface IL‐1*α* (as opposed to the secreted form). Blocking cell surface IL‐1*α* reduces senescence related secretion of IL‐6 and IL‐8 (Orjalo et al. 2009). The capacity for the entire response to catecholamines being driven by IL‐1*α* signaling is evident, but how these cytokines interact in skeletal muscle is not entirely clear. Some evidence suggests that IL‐1*α* inhibits ryanodine receptors in skeletal muscle, which may implicate IL‐1*α* as a mediator of critical illness‐induced muscle weakness, but whether this response is triggered or enhanced by catecholamines remains to be determined (Friedrich et al. 2014).

The functional significance of CXCL1 secretion in conditions of stress, when catecholamines are high, has been rarely investigated. However, besides its well‐recognized role as a neutrophil chemoattractant, CXCL1 also stimulates progenitor stem cell mobilization from bone marrow (Li et al. 2014), has neuroprotective properties (Omari et al. 2009), suppresses myocarditis in autoimmune inflammatory heart disease (Bachmaier et al. 2014), contributes to host defense in sepsis (Jin et al. 2014) and has positive influences on fatty acid oxidation and muscle oxidative capacity (Pedersen et al. 2012). Although CXCL1 is often described as the mouse homolog for human IL‐8, it has overlapping functions with the human form of CXCL1 and CXCL3, all considered orthologs of CXCL1. We hypothesize that CXCL1 may play a complementary role to the stress‐hormone activities of IL‐6 (Welc and Clanton 2013) and these may, in some conditions, be orchestrated by stress‐induced endocrine networks of catecholamines, IL‐1*α*, and IL‐6.

The lack of significant cytokine secretion in response to the DFO/EPI/NOREPI combination from the primarily fast‐twitch EDL muscles was somewhat expected. Multiple studies have demonstrated a lower beta‐adrenergic receptor density in predominantly type II muscles than in type I (Williams et al. 1984; Martin et al. 1989; Jensen et al. 1995). Although, exercise has been shown to elicit IL‐6 release from type II skeletal muscle (Hiscock et al. 2004). The results of this study suggest that this exercise‐induced response seen by others may be due to other exercise‐related factors, such as contraction, calcium concentration, increased core temperature, and glycogen depletion, rather than mediation by circulating catecholamines. However, we are cautious to dismiss the potential for catecholamine‐mediated potentiation in cytokine secretion occurring during exercise when so many signaling paths are stimulated concurrently. Unexpectedly, the more beta‐adrenergic receptor dense solei muscles also failed to generate increased cytokine secretion in response to DFO/EPI/NOREPI over DFO alone, suggesting that the signal transduction initiated by DFO may have already potentiated MAPK‐ and JNK‐mediated paths. Thus, a cytokine secretory effect of EPI and NOREPI alone cannot be ruled out due to the obscuring effect of DFO. Unfortunately, the oxidative issues in vitro still remain a confounding problem.

We attempted to improve the fidelity of the dose response relationship to catecholamines in vitro by using DFO to prevent autoxidation of EPI, theoretically making EPI more stable in solution due to the metal chelating properties of DFO (Ryan et al. 1993). As stated above, we observed that DFO alone was a potent stimulus for both IL‐6 and CXCL1 in the absence of catecholamines and that no other cytokines were appreciably affected by this treatment. Interestingly, DFO is naturally found as a siderophore on the surface of multiple bacterial species, which use DFO like substances to chelate and take up free iron into their cells (Müller and Raymond 1984). In the absence of bacteria, the DFO alone can activate p38, ERK1/2, MAPK, and JNK in intestinal epithelial cells, and these have been shown to induce IL‐6 secretion (Choi et al. 2004; Markel et al. 2007). One other potential mechanism is through activation of the unfolded protein response which has been shown to be activated at relatively low doses of DFO in PC12 cells (Yoo et al. 2008). We (Welc et al. 2013) and others have previously shown that activation of the UPR is a potent stimulus for IL‐6, and it has recently been shown in endothelial cells to be a stimulus for CXCL1 (Gargalovic et al. 2006).

The low sensitivity of isolated skeletal muscle to catecholamine exposure questions the biological relevance of this hormonal response in muscle. However, this lack of sensitivity parallels the findings of nearly all other tissues studied in isolated buffer solutions. For example, classic studies of EPI sensitivity of aortic strips (Furchgott and Bhadrakom 1953) demonstrate less than 10% peak contractile force at about 100‐200 ng/mL, comparable to our EPIHIGH dose. In the isolated rat heart, increases in dV/dt and heart rate require about 100 ng/mL in the oxygenated buffer to see any significant changes at all (Zausig et al. 2010). We can contrast this with cardiac studies in vivo in the rat, in which the EC50 for the effect of epinephrine on dP/dT is 0.34 ng/mL, a greater than 100‐fold increase in EPI sensitivity going from in vitro heart to in vivo conditions (Maslov et al. 2015). For IL‐6 secretion, the same apparent effect was shown by Frost and Lang (2005). In response to in vivo infusion of EPI they observed significant elevations in both muscle mRNA for IL‐6 and for IL‐6 protein at a plasma concentrations of 1.2 ng/mL EPI, whereas the EC50 in isolated myoblasts was roughly 10‐fold higher. These comparisons provide an insight into the difficulties of equating in vivo concentrations with the biological sensitivity, in vitro*,* when it comes to studies with EPI. The phenomenon may be due to poor EPI stability in plasma versus physiological buffers in the absence of thiols like glutathione that are active in blood and plasma (Pettersson et al. 1980). Obviously, from this study, DFO treatment did not solve the problem. Therefore, it remains our perception that the low sensitivity of this secretory response to catecholamines does not rule out its biological relevance in vivo.

In summary, our data are consistent with a strong CXCL1 response to EPI stimulation that parallels changes seen in IL‐6 and accompanies alterations in IL‐1*α*. Whether this is physiologically relevant in the intact organism during catecholamine secretion associated with acute stress, exercise, or illness remains an open question. We speculate that together with IL‐6, in conditions of stress, catecholamines may function as an efferent arm of a hormonal reflex encompassing not only IL‐6 but also CXCL1 that assists organisms in overcoming the challenges of extreme stress exposure.

## Conflict of Interest

None declared.
